# Neurodegeneration Through the Lens of Bioinformatics Approaches: Computational Mechanisms of Protein Misfolding

**DOI:** 10.3390/ijms262211021

**Published:** 2025-11-14

**Authors:** Mubashir Hassan, Saba Shahzadi, Ahmed A. Moustafa, Andrzej Kloczkowski

**Affiliations:** 1 The Steve and Cindy Rasmussen Institute for Genomic Medicine at Nationwide Children’s Hospital, Columbus, OH 43205, USA; saba.shahzadi@nationwidechildrens.org; 2 School of Psychology, Faculty of Society and Design, Bond University, Gold Coast, QLD 4229, Australia; amoustaf@bond.edu.au; 3 Department of Human Anatomy and Physiology, Faculty of Health Sciences, University of Johannesburg, Johannesburg 2028, South Africa; 4 Department of Pediatrics, The Ohio State University, Columbus, OH 43205, USA; 5 Department of Biomedical Informatics, The Ohio State University, Columbus, OH 43210, USA

**Keywords:** protein aggregation, databases, computational methods, bioinformatics, neurodegenerative diseases

## Abstract

Protein and peptide aggregation has become a prominent focus in biomedical research due to its critical role in the development of neurodegenerative diseases (NDs) and its relevance to industrial applications. Neurodegenerative disorders such as Alzheimer’s disease (AD), Parkinson’s disease (PD), Huntington’s disease (HD), and Amyotrophic Lateral Sclerosis (ALS) are closely associated with abnormal aggregation processes, highlighting the need for a deeper understanding of their molecular mechanisms. In recent years, a wide range of computational methods, bioinformatics tools, and curated databases have been developed to predict and analyze sequences and structures that are prone to aggregation. These in silico approaches offer valuable insights into the underlying principles of aggregation and contribute to the identification of potential therapeutic targets. This review provides a concise overview of the current bioinformatics resources and computational techniques available for studying protein and peptide aggregation, intending to guide future research efforts in the field of neurodegenerative disease modeling and drug discovery.

## 1. Introduction

The human proteome comprises 20,000–25,000 proteins with different residue lengths, structures, and functions [1,2]. Proteins are the most well-known targets in biomedical applications and drug design [3,4,5]. Protein aggregation is a process that results in misfolded proteins assembling into insoluble aggregates [6,7], which leads to various disorders, including Amyotrophic Lateral Sclerosis (ALS), Alzheimer’s disease (AD), Parkinson’s disease (PD), and Prion diseases [8,9,10]. The formation of intracellular and extracellular proteinaceous deposits is considered the primary cause of many illnesses, despite various risk factors, including aging, environmental factors, and genetic abnormalities [5,11,12].

The sequence of amino acids determines the three-dimensional protein structure and dictates its specific shape and chemical environment, which are related to its functional capacity [13,14]. Proteins fold into stable, functional 3D shapes by minimizing their free energy through favorable molecular interactions. The native protein conformation is crucial for the biochemical functions of proteins, including enzymatic activities, specific binding properties, and signaling capabilities [15,16]. In protein structural biology, intrinsically disordered regions (IDRs) and intrinsically disordered proteins (IDPs) play crucial roles in cellular biology, challenging the conventional structure-function paradigm of proteins. IDPs and IDRs deviate from the traditional protein structure-function relationship by existing as collections of rapidly interconverting conformations, rather than having a single, unique structure [17]. Specific proteins and regions contain high levels of disorder-promoting amino acids, including Ala, Arg, Gly, Gln, Glu, Lys, Pro, and Ser. It has been observed that IDPs and IDRs play a crucial role in neurodegeneration across a range of diseases characterized by protein aggregation and neuronal death [17].

Protein aggregation is a hallmark of many neurodegenerative disorders, driven by several molecular mechanisms that disrupt normal protein folding and stability. One major contributor is proteolytic cleavage, which generates truncated protein fragments with exposed hydrophobic regions that are prone to misfolding and aggregation, such as the cleavage of amyloid precursor protein (APP), leading to amyloid-β accumulation in AD [18]. Point mutations also play a critical role by altering amino acid sequences in ways that destabilize native conformations or promote β-sheet formation, as observed in familial forms of Parkinson’s and Huntington’s disease [19]. Additionally, post-translational modifications (PTMs), including phosphorylation, ubiquitination, acetylation, and glycosylation, can significantly affect protein solubility, charge distribution, and structural dynamics. For example, hyperphosphorylation of tau protein disrupts its normal function and promotes aggregation into neurofibrillary tangles [20].

In neurodegeneration, intrinsically disordered Aβ peptides aggregate to create amyloid plaques, while hyperphosphorylated tau protein aggregates to form neurofibrillary tangles in AD [19]. These formations are linked to neurodegeneration and cognitive impairment [21]. Lewy bodies, indicative of Parkinson’s disease etiology, are also formed by the highly disordered protein α-synuclein [22]. Aggregates with extended intrinsically disordered areas of the Transactive response DNA-binding protein 43 (TDP-43) and Fused in sarcoma (FUS) proteins are associated with frontotemporal dementia and ALS [22].

Depending on the thermodynamic stability of the protein, aggregation often results from chemical or physical breakdown. The main factor contributing to protein aggregation has been shown to be a decrease in free surface energy, caused by the extraction of hydrophobic residues from solvent interactions [23]. Moreover, colloidal stability is critical in controlling protein aggregation, particularly in pharmaceutical and biomedical applications [24]. It describes the capability of protein solutions to remain dispersed without sedimentation or aggregation of the particles, which is crucial for ensuring the efficacy and safety of protein therapeutics. The colloidal stability directly impacts the aggregation propensity of the protein formulations. Aggregation in proteins is known to reduce or even eliminate their biological function concurrently. Sometimes, such changes in structure can also induce undesirable immunological reactions in patients [25]. The potential for these risks, especially concerning protein aggregation upon interaction, is minimized because of colloidal stability. In neurodegenerative diseases, the primary factor involved in pathology is the aggregation of misfolded proteins. Examples of proteins include α-synuclein in PD and β-amyloid in AD, where extensive aggregations contribute to neurotoxicity.

The ability of such aggregates to disrupt cellular functions and promote neuronal death means that high colloidal stability can be an essential factor in reducing the potential for protein aggregation in NDs [4,26,27]. Previous research has shown that when aggregates grow above the solubility limit, it results in the formation of insoluble aggregates [28]. Protein optimization has become increasingly important in the development of biotherapeutic medications. This process focuses on enhancing protein stability and solubility, while simultaneously reducing viscosity and aggregation, all of which are critical factors for drug efficacy and safety. By addressing these characteristics, optimized proteins can offer improved pharmacokinetics and pharmacodynamics, leading to more effective treatments [24,29,30]. However, a biopharmaceutical aggregation propensity may influence its solubility and viscosity in liquid formulations [31,32]. This review examines the computational approaches that can aid in predicting and enhancing the understanding of protein aggregation principles associated with neurodegenerative diseases.

## 2. Protein Aggregations and Neurodegenerative Diseases (NDs)

Many neurodegenerative diseases, including Alzheimer’s disease, Parkinson’s disease, and Huntington’s disease, are characterized by protein aggregation [7]. In these disorders, specific proteins within neurons, such as β-amyloid and tau in Alzheimer’s disease, α-synuclein in Parkinson’s disease, and huntingtin in Huntington’s disease, begin to misfold and lose their regular structure, forming aggregates that can disrupt normal cell function and lead to neuronal damage and death [33]. There are two main hypotheses about the role of protein aggregation in NDs: (i) The toxic gain-of-function hypothesis: This hypothesis proposes that the aggregated proteins themselves are harmful to neurons. Protein aggregates can interfere with normal cellular activities, damage cell membranes, and disrupt the function of other proteins. One key explanation for their harmful effects is the loss-of-function hypothesis, which suggests that as proteins aggregate, they reduce the availability of properly folded, functional proteins. This depletion can impair essential cellular functions and may ultimately lead to the death of neurons [34,35]. (ii) The second hypothesis is that protein aggregation is used as a mechanism to overcome stress in the organism [36].

### 2.1. Protein Aggregation and Alzheimer’s Disease (AD)

Protein aggregation is considered an AD characteristic, and a couple of proteins, amyloid beta (Aβ) and tau, clump together in the brain, forming plaques and tangles toxic to neurons [3,37]. The term “Aβ” refers to peptides containing 36–43 amino acids, which are the primary building blocks of the amyloid plaques in AD patients’ brains. Aβ is a protein fragment separated from a larger protein called amyloid precursor protein (APP) [38]. Aβ plaques are found extracellularly in the brain parenchyma and within the walls of blood vessels [39]. However, in AD, Aβs clump together to form amyloid plaques, which disrupt communication between neurons and impair brain function. Moreover, soluble Aβ oligomers are particularly toxic, impairing synaptic function and plasticity, which are crucial for learning and memory [40]. Aβ plaques induce oxidative stress, disrupt calcium homeostasis, and activate apoptotic pathways, leading to neuronal death. Aβ aggregates activate microglia and astrocytes, leading to chronic neuroinflammation. The release of pro-inflammatory cytokines exacerbates neuronal damage [41].

Tau, a microtubule-associated protein, becomes hyperphosphorylated, leading to its dissociation from microtubules [42]. Hyperphosphorylated tau aggregates into paired helical filaments and neurofibrillary tangles within neurons. Tau protein typically helps stabilize structures inside nerve cells called microtubules [43]. In AD, tau becomes abnormally modified and clumps together to form tangles. Tangles disrupt the transport of nutrients and other vital materials within neurons, ultimately leading to cell death. There are different functions associated with the tau protein upon aggregation [44]. The loss of tau’s microtubule-stabilizing function disrupts axonal transport, affecting the delivery of nutrients and organelles within neurons. The accumulation of tau tangles impairs cellular functions and ultimately leads to neuronal apoptosis [45].

### 2.2. Protein Aggregation and Parkinson’s Disease (PD)

Protein aggregation, specifically of the α-synuclein protein, is a key element in the development of PD [46]. It has been observed that α-synuclein misfolds and clumps together to form Lewy bodies, abnormal aggregates that are a hallmark characteristic of PD. The α-synuclein is composed of 140 residues, which lack both cysteine and tryptophan amino acids, whereas its N-terminus is positively charged and rich in lysine amino acids [47]. In the healthy brain and central nervous system (CNS), wild-type α-synuclein forms soluble monomers and is thought to facilitate physiological activity in presynaptic terminals [48]. In vitro studies indicate that α-synuclein mutations like A30P and A53T, which cause early-onset familial PD, result in oligomers rather than β-sheet aggregated fibrils. The non-amyloid component (NAC) domain of α-synuclein is a 12-amino-acid hydrophobic sequence that plays a crucial role in the protein’s transformation from a soluble monomer to an oligomer, protofibril, and fibril. Mutant variants of α-synuclein lead to faster fibril production than wild-type proteins. Individuals with A30P and A53T α-synuclein point mutations are more susceptible to α-synuclein aggregation and toxicity, resulting in an earlier onset of symptoms [49,50].

### 2.3. Protein Aggregation and Huntington’s Disease (HD)

**Figure 1:**
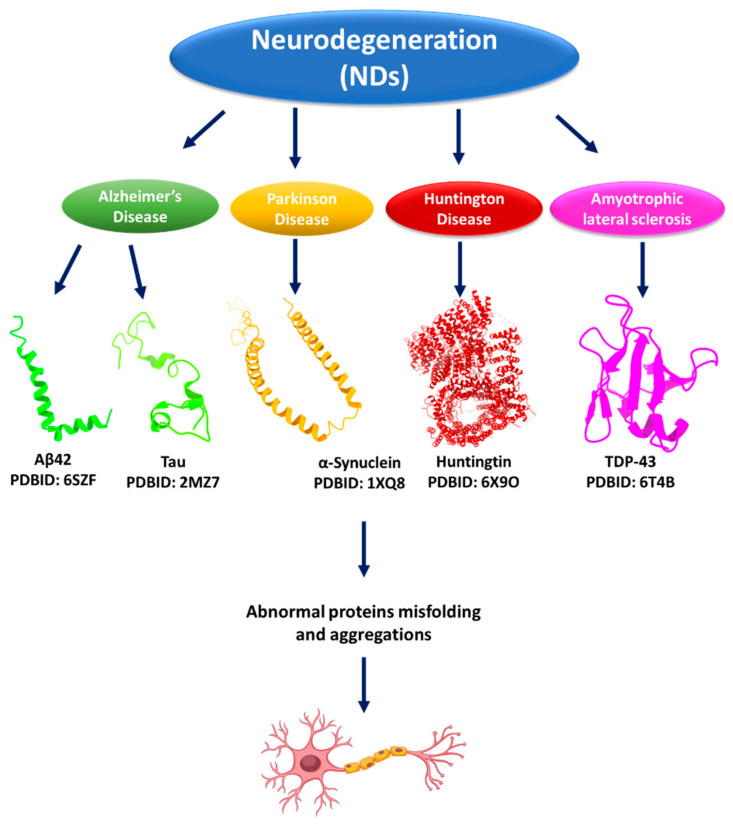
Protein aggregation and neurodegenerative diseases (NDs). Aβ42 primarily affects pyramidal neurons in regions such as the hippocampus and cortex, leading to synaptic dysfunction and contributing to disruptions in central nervous system (CNS) pathways relevant to Alzheimer’s disease therapy development. Tau protein, predominantly expressed in CNS neurons, plays a key role in stabilizing microtubules for intracellular transport and neuronal function, with limited expression in oligodendrocytes, highlighting its importance in maintaining neuronal integrity. α-synuclein is primarily localized in excitatory and select inhibitory neurons across critical CNS regions, where it regulates synaptic transmission and is central to the pathology of Parkinson’s disease and therapeutic targeting. HTT protein, widely distributed in CNS neurons, supports motor and cognitive functions and is closely linked to neuronal development, making it a focal point for Huntington’s disease research. In ALS, TDP-43 is a nuclear RNA-binding protein that aberrantly accumulates in the cytoplasm of motor neurons, forming toxic aggregates that impair RNA metabolism and protein homeostasis. Its mislocalization and aggregation are key pathological features in the majority of ALS cases, underscoring its significance in disease progression and therapeutic exploration.

Huntington’s disease (HD) is another neurodegenerative disorder caused by huntingtin (HTT) protein (3144 amino acids) aggregation in human brain nerve cells [51]. The HTT protein and its aggregation are critical to the development of HD. The mutant HTT (mHTT) self-aggregates into both soluble oligomers and insoluble fibrils, thereby affecting vital cellular processes such as cell quiescence and cell death. The disease-causing mutant HTT has a larger polyglutamine (polyQ) domain due to an increase in glutamine-encoding CAG repeats [52]. Healthy individuals typically have 6 to 35 CAG repeats, while those with more than 39 are more likely to develop HD. The number of repeats shows a negative correlation with HD age of onset. High repeat counts can lead to juvenile-onset HD, which progresses faster than adult-onset HD. The polyQ expansion mutation results in a harmful gain-of-function mutation in the protein, thereby increasing its aggregation potential. HD is characterized by the production of mHTT aggregates and inclusion bodies within cells, leading to cell quiescence and eventual cell death [52]. The polyQ domain in HTT has significant implications for the conformational dynamics of huntingtin. Generally, the expansion of polyglutamines in this protein leads to conformational changes that cause aggregation and toxicity. Notably, with the increase in the length of the polyQ repeat, the protein’s tendency to misfold into aggregates also increases, disrupting cellular functions in neurons—a hallmark of pathology in HD [53]. Studies have indicated that the toxic properties of mHTT are strongly modulated by the length of the polyQ stretch. The polyQ region may alter the structural stability of HTT, allowing it to adopt conformations that tend to aggregate into neurotoxic forms. Indeed, several studies have suggested that these expanded sequences cause misfolding, which initiates neurodegenerative processes, reflecting an essential pathological mechanism underlying HD [53] (Figure 1).

### 2.4. Protein Aggregation and Amyotrophic Lateral Sclerosis (ALS)

Protein aggregation plays a central role in the pathogenesis of Amyotrophic Lateral Sclerosis (ALS), a progressive neurodegenerative disorder affecting motor neurons. In nearly 97% of ALS cases, the RNA-binding protein TDP-43 mislocalizes from the nucleus to the cytoplasm, where it forms insoluble aggregates that disrupt cellular homeostasis and contribute to neurotoxicity [54,55]. Similarly, mutations in superoxide dismutase 1 (SOD1) lead to the formation of misfolded protein species that aggregate, triggering oxidative stress and mitochondrial dysfunction [55]. These aggregation events are not merely byproducts of disease but active drivers of neuronal degeneration [56]. Targeting these aggregation-prone proteins, whether through small molecules, antisense oligonucleotides, or immunotherapy, represents a promising therapeutic strategy; however, challenges remain in early detection and patient-specific variability. Understanding the molecular mechanisms behind protein misfolding and aggregation is therefore crucial for developing effective interventions in ALS [57] (Figure 1).

Table 1 presents curated resources for studying protein aggregation, organized by disease-specific and general-purpose databases and tools. This classification helps researchers identify relevant platforms for investigating aggregation mechanisms in neurodegenerative diseases such as AD, PD, HD, and ALS, as well as broader contexts.

## 3. Protein Aggregation Resources

Databases are online resources where information is stored and accessed for research and commercial purposes [58]. Several online databases are available to serve the research community working on protein aggregation and therapeutic interventions (see Table 2) [59]. Fibril_one is an amyloidogenic protein database composed of 250 mutations of 22 proteins accompanied by 50 experimental conditions [60]. Fibril_one contains information about fibril formation and is annotated based on extensive literature searches. It is linked with different databases such as GenBank [61], SWISS-PROT [62], and PDB [63], respectively.

ZipperDB (https://services.mbi.ucla.edu/zipperdb/ (accessed on 9 November 2025)) is an additional online resource that provides aggregation profiles of 76 genomes [64]. It contains predictions for protein fibril-forming sites identified by the 3D Profile Method. This method involves searching through more than 20,000 potential protein sequences for regions with a high tendency for fibrillation, which could result in a “steric zipper” composed of two self-complementary β-sheets that give rise to the spine of an amyloid fibril [65]. The assembly of experimentally known amyloid-forming hexapeptides investigated using Fourier-transform infrared spectroscopy (FTIR), dye binding, and electron microscopy is cataloged in WALTZ-DB (http://waltzdb.switchlab.org/ (accessed on 9 November 2025)) [66]. The core predictive capabilities of ZipperDB rely on the 3D Profile Method. This algorithm threads each six-residue peptide from a given protein sequence onto the crystal structure of a specific fibril-forming peptide, NNQQNY, obtained from the Sup35 prion protein of Saccharomyces cerevisiae (*S. cerevisiae*). The energetic compatibility of this fit is assessed using the RosettaDesign program, allowing for the identification of peptides that demonstrate a high propensity to form fibrils. ProADD is a database on protein aggregation diseases that was created to bring all the information together on one platform for users’ simple access [67]. This database enables the categorization of protein aggregation illnesses through structural and sequence analysis, allowing for the identification of protein aggregation patterns within the dataset. The database contains information on over 600 proteins associated with various protein aggregation diseases, serving as a valuable resource for researchers. Proteins are categorized by disease associations and their structural properties, aiding in analyzing protein behavior in disease conditions [67]. The algorithm that underpins ProADD works by methodically gathering and classifying information on proteins implicated in aggregation disorders. Central to this approach is categorizing proteins according to their structural and sequence features. It provides scientists with access to a wealth of data, facilitating in-depth examinations of protein regions prone to aggregation and potentially linking their characteristics to disease processes [67].

AmyLoad database collects amyloidogenic and non-amyloidogenic sequence fragments from all possible primary resources and provides detailed information about each fragment [68]. The algorithm associated with AmyLoad, known as Amyloid IQ, plays a crucial role in analyzing amyloid imaging data. Amyloid IQ is an advanced software for analyzing amyloid PET images to measure amyloid load (AβL), a vital marker of Alzheimer’s disease. It works in conjunction with AmyLoad, which focuses on amyloidogenic protein fragments, providing a database and tools for amyloid research [69]. The AmyLoad algorithm offers a sophisticated technique for identifying and measuring Aβ oligomers and fibrils, which influence protein aggregation. To function correctly, proteins typically require specific three-dimensional conformations, and they can assemble and misfold in pathological situations. The AmyLoad algorithm helps detect these formations by using improved imaging methods that measure the amount of amyloid aggregates in the brain and determine their presence [70]. Another extensive database covering precursor proteins and the areas prone to aggregation is called AmyPro [71]. In addition to providing information about these proteins, AmyPro offers phylogenetic annotations of proteins and their functions in the amyloid state, as well as links to other databases and research references. As a result, the amyloidogenic sequence segments within the associated protein structures are detected instantly. AmyPro’s underlying algorithm was primarily developed to identify amyloidogenic regions in protein sequences. It utilizes machine learning methods to analyze amino acid sequences and determine the likelihood that specific protein segments will form amyloid fibrils [72].

The Curated Protein Aggregation Database (CPAD: https://web.iitm.ac.in/bioinfo2/cpad2/ (accessed on 9 November 2025)) is an extensive database that compiles findings from scientific community-conducted experimental research aimed at understanding protein/peptide aggregation [73]. The information included in CPAD has been combined with other data, including peptides of varying length that form amyloid fibrils, hexapeptides that form amyloid fibrils and whose crystal structures are available in PDB, and experimentally verified regions of amyloidogenic proteins that are prone to aggregation [73]. A new version of CPAD 2.0 has been launched, which includes aggregation-related protein structures [74].

AmyloBase (http://bioserver2.sbsc.unifi.it/amylobase/pages/view.html (accessed on 9 November 2025)) is a database that collects data on protein aggregation from kinetics experiments [75]. AmyloBase gathers and arranges monoclonal light chain sequences that are particularly linked to AL amyloidosis, a disorder in which aberrant protein aggregates cause tissue damage. To identify and examine sequence features that facilitate the development of amyloid fibrils, this database comprises more than 2200 sequences from multiple myeloma, AL amyloidosis, and other plasma cell diseases [76]. AMYPdb is another database that collects structural information on amyloidogenic proteins [77]. The core algorithm of AMYPdb, called Salsa, is designed to predict the aggregation propensities of both single and multiple protein sequences based on their physicochemical properties (http://amypdb.genouest.org/e107_plugins/amypdb_project/project.php (accessed on 9 November 2025)). Salsa calculates probability indexes indicating potential ‘hot spots’ within protein sequences likely to drive amyloid formation. By identifying these regions, the algorithm enables researchers to understand where aggregation is most likely to occur within a given protein sequence [78]. PDB_Amyloid database (https://pitgroup.org/amyloid/ (accessed on 9 November 2025)) contains entries on both amyloid and globular proteins with amyloid-like substructures [79]. PDB_Amyloid compiles a list of amyloid structures exhibiting a characteristic cross-β sheet conformation essential for amyloid formation. These structures are crucial in various diseases associated with protein misfolding and aggregation. AL-Base database collects information about amyloidogenic immunoglobulin light chain sequences derived from patients with AL amyloidosis [80].

The Aggrescan3D (A3D: https://biocomp.chem.uw.edu.pl/A3D2/MODB (accessed on 9 November 2025)) database includes human protein structures predicted by AlphaFold, with a special emphasis on their aggregation characteristics. Each amino acid is structurally corrected through aggregation values (A3D score) determined by the A3D algorithm, utilizing 3D atomic models [81,82]. An A3D database provides a comprehensive protein structure-based analysis of aggregation propensity. The A3D database offers easy-to-use graphical user interfaces for visualizing protein structures. It also enables the users to validate the effect of mutations on protein solubility and stability [83]. The latest update expands the database’s coverage to encompass over 160,000 proteins, resulting in more than 500,000 structural aggregation predictions across twelve key model organisms, which were selected to represent a broad evolutionary spectrum. This extension aims to bridge gaps in understanding protein aggregation beyond humans, allowing comparative and evolutionary studies that can leverage proteomic diversity [84]. The Cryptic Amyloidogenic Areas Database (CARs-DB) (http://carsdb.ppmclab.com/ (accessed on 9 November 2025)) focuses on intrinsically disordered proteins (IDPs), which, compared to traditional amyloid areas enclosed within globular proteins, tend to aggregate less. But it also seems that their existence is linked to diseases like AD or cancer. The CARs-DB database contains precomputed predictions for every CAR identified in the IDPs that have been deposited in the DisProt database, comprising more than 8900 distinct CARs found in 1711 IDRs [85,86,87].

PASTA is an online server that predicts protein aggregation based on sequence. In 2007, the initial version of PASTA server was proposed. PASTA 1.0, the Prediction of Amyloid Structure Aggregation server, emerged as a pioneering computational tool that predicts the most aggregation-prone portions in protein sequences by modeling the stability of cross-beta structures [88]. PASTA 1.0 was designed to predict amyloid-forming regions from protein sequences by analyzing pairwise energy potentials between residues. The central concept is that the amyloid cross-beta structure, held together by extended hydrogen bonds along the fibril axis, can be predicted from patterns of β-sheet pairings observed in globular proteins. The algorithm uses a statistical energy function derived from datasets of globular protein structures, identifying which pairs of residues are likely to be found facing each other in β-sheets with either parallel or antiparallel arrangements [89]. This approach effectively translates the principles of beta-sheet formation in normal proteins into the prediction of pathological amyloid fibril regions. Ultimately, PASTA 1.0 has deepened our understanding of amyloid formation mechanisms and continues to make significant contributions to research on protein misfolding diseases and therapeutic targeting. In 2014, a new version of PASTA 2.0 was launched, which includes prediction of protein secondary structure and intrinsic disorder to increase the accuracy of predicting aggregation. The PASTA 2.0 (http://protein.bio.unipd.it/pasta2/ (accessed on 9 November 2025)) energy function assesses the potential stability of cross-β pairings between distinct sequence segments [89]. The PASTA algorithm utilizes a pairwise energy potential method to assess the molecular interactions between distinct protein sequence regions. Thanks to this architecture, the algorithm can successfully predict amyloid fibril locations from input protein sequences [89].

**Table 2:** Computational resources for protein aggregation.

Resources	Classification	Functions	Ref
Fibril_one	Database	Fibril_one database serves as a specialized resource for managing and analyzing data on fibrils, particularly in biological and biochemical research.	[60]
ZipperDB	Algorithm (with Database)	ZipperDB employs a novel algorithm that utilizes structural information to predict fibril-forming segments within proteins.	[64]
WALTZ-DB 2.0	Database	WALTZ-DB 2.0 serves as a significant resource for the characterization of short peptides based on their ability to form amyloid fibers	[66]
ProADD	Database	The ProADD database focuses on protein aggregation diseases and provides valuable information on the underlying mechanisms of protein aggregation in Alzheimer’s and Parkinson’s diseases.	[67]
AmyLoad	Database	AmyLoad is designed for amyloidogenic protein fragments and protein aggregation, with a focus on their significance in Alzheimer’s disease.	[68]
AmyPro	Database	AmyPro is an open-access resource specifically designed to collect and analyze proteins with validated amyloidogenic regions.	[71]
CPAD 2.0	Database	CPAD 2.0 focuses on various aspects of protein aggregation, including mechanistic, kinetic, and structural information, which are crucial for understanding protein-related diseases.	[74]
AmyloBase	Database	The primary function of the AmyloBase database is to facilitate the organization, retrieval, and analysis of data related to amyloids.	[75]
AMYPdb	Database	AMYPdb is a specialized database dedicated to amyloid precursor proteins.	[77]
PDB_Amyloid	Database	PDB_Amyloid provides access to a diverse range of amyloid structures, which can be explored for research and educational purposes.	[79]
AL-Base	Database	The AL-Base database plays a pivotal role in studying and understanding light chain sequences associated with amyloidosis and related diseases.	[80]
A3D	Algorithm (with Database)	A3D is to facilitate the prediction of protein aggregation based on its structural attributes.	[83]
CARs-DB	Database	CARs-DB is a pivotal resource for protein chemistry, specifically in understanding the amyloidogenic properties of intrinsically disordered proteins and their links to various diseases.	[85]
PASTA 2.0	Algorithm	PASTA 2.0 serves as a comprehensive tool for researchers studying protein aggregation. Its primary function is to analyze protein sequences and assess their potential for aggregation.	[89]

## 4. In-Silico Techniques to Investigate Protein Aggregation

In recent decades, many computational studies on protein aggregation have been done [90]. In research on protein aggregation, three fundamental computational methods are employed: (i) assessing the tendency for aggregation, (ii) predicting the kinetics of aggregation, and (iii) using molecular dynamics simulations [59,91].

### 4.1. Protein Sequence and Aggregation

Sequence-based methods for predicting protein aggregation include examining the physicochemical characteristics of amino acids, sequence patterns, statistically determined propensity values, knowledge-based scoring functions, residue-residue contact potentials, secondary structure propensities, and threading [92,93]. The sequence-based methods commonly used to examine linear sequences are based on pattern matching, which includes amyloidogenic pattern matching. Positional scanning mutagenesis has been utilized in one of these investigations to target the STVIIE peptide sequence and identify the sequence patterns of hexapeptides that combine to generate fibrils resembling amyloid [94].

Identifying linear polypeptide sequences was the foundation for constructing algorithms to predict protein aggregation [95]. Their design is based on the idea that short, distinct sequential segments, typically hydrophobic and low in net charge, cause protein aggregation. The phenomenological class of algorithms, on the other hand, is associated with experimental data that establishes the determinants of aggregation [96]. The best examples of phenomenological algorithms, which rationalize factors discovered through experimentation that influence protein aggregation, are AGGRESCAN and Zyggregator [97,98]. The second category relies on theoretical evaluations of sequence features known to be implicated in aggregation. That includes algorithms such as TANGO, PASTA 2.0, FoldAmyloid, Waltz, and Amyloid mutants. These tools assess the potential of a sequence to form the topologically constrained conformations typical of amyloid-like states, protein packing density, residue pattern and composition, and the propensity of a sequence to form a specific aggregation-prone conformation [99].

Additionally, an increasing number of machine learning (ML)-dependent techniques are being developed to predict protein aggregation. Machine learning algorithms, specifically neural networks, are employed to perform feature extraction on sequential data and identify highly correlated patterns that contribute to an aggregated output [100]. ML models achieve superior or equivalent performance compared to traditional methods, with notable examples including APPNN [101] and netCSSP [102]. Additionally, some consensus algorithms combine and weight the outputs of several predictors to provide a single forecast; notable examples of these algorithms include AMYLPRED 2 and MetAmyl [103,104].

### 4.2. Protein Aggregation Using Amino Acid Fundamental Characteristics

Amino acid properties related to hydrophobicity, charge, size, and other specific side-chain interactions represent critical parameters in protein aggregation. The interplay of these factors determines not only the stability of the protein structure but also its susceptibility to misfolding and aggregation, with profound implications for protein function and disease development [105,106]. Aggregation-prone areas are identified by several physicochemical features of amino acids, including β-sheet propensity, hydrophobicity, size, surface area, charge, aromaticity, and contact frequency. The best example of algorithms that utilize the amino acid aggregation-propensity scale derived from in vivo experiments on amyloidogenic proteins is AGGRESCAN [98].

Likewise, the Zyggregator algorithm employs a feature-based analysis to predict protein aggregation, utilizing a set of characteristics derived from the amino acid sequence. These features include hydrophobic properties, charge distribution, secondary structure propensities, and the presence of specific gatekeeper residues [107]. The WALTZ technique employs a hybrid methodology that integrates a position-specific pseudo-energy value derived from modeled structures with a position-specific score matrix generated from amyloidogenic peptides and amino acid physicochemical properties [108]. ANuPP consists of nine logistic regression models, each trained independently on distinct sets of amyloidogenic peptides, capturing the variability in nucleation, diffusion, and fibrillation mechanisms of aggregate formation [109].

### 4.3. Protein Secondary Structure and Aggregation

The secondary structure propensity is a crucial factor in protein aggregation, influencing stability and the likelihood of aggregation [91]. Protein chains fold into distinct secondary structure elements, such as α-helices and β-sheets, which are stabilized by hydrogen bonds between backbone atoms [110]. The regular patterns formed in these structures are essential for the overall stability and functionality of the protein. Especially, β-sheets are highly represented in aggregated states such as amyloid fibrils. These structures form a stable scaffold through which intermolecular interactions between proteins can occur, thereby leading to aggregation. Aggregation-prone regions (APRs) of proteins, typically enriched in hydrophobic residues, align in extended β-sheet structures that play a crucial role in the aggregation pathway. This tendency leads to the accumulation of misfolded proteins, a common characteristic of neurodegenerative diseases like Alzheimer’s disease and PD [111,112]. The finest example of such an algorithm is TANGO, which estimates the likelihood that a segment will form β-strand-mediated aggregates using potential functions obtained both empirically and statistically [113].

Furthermore, TANGO examines the odds of having various secondary structure states, including coil, turn, β-sheet, and α-helix. Similarly, to forecast protein aggregation, the SecStr and NetCSSP algorithms assess conformational transitions from other secondary states to β-sheet [102,114]. It has recently been shown that the β-content of the monomer determines the aggregation tendency, and a higher β-content correlates with faster protein aggregation [115].

### 4.4. Protein Aggregation Based on Amino Acids’ Interactive Profiles

The interactions between two amino acids, known as residue pairs, are fundamental to the structure and function of proteins. Protein architectures and functions can be predicted by analyzing these pairings. This approach evaluates contact predictions to uncover the underlying patterns of protein folding and stability [91]. The cross-β spine of amyloid fibrils often features a double β-sheet, each of which consists of parallel segments stacked in register. The two sheets are joined by interdigitated side chains and axial hydrogen bonds, forming a tightly self-complementing steric zipper. Moreover, aromatic residues that form stacking and ladders of hydrogen bonds, including Asn, Gln, Thr, and Ser, contribute to extra stability. Some approaches for predicting protein aggregation heavily rely on these residue-residue interactions [116], with examples such as PASTA2 and BETASCAN that use residue-residue probabilities and scoring functions for β-sheet hydrogen bond formation and contacts derived from protein structure databases [90].

### 4.5. Structure-Based Techniques

Structure-based techniques have emerged as essential tools for better studying and understanding protein aggregation, enabling researchers to identify, predict, and even inhibit aggregation through rational design [117]. Structure-based techniques utilize the three-dimensional structures of proteins to analyze and predict the propensity for protein aggregation. They focus on identifying aggregation-prone regions (APRs) within proteins, which are short segments that are likely to participate in the formation of aggregates. Techniques such as X-ray crystallography, nuclear magnetic resonance (NMR) spectroscopy, and cryo-electron microscopy provide detailed structural insights that inform these predictions [72,95,118]. The Spatial Aggregation Propensity (SAP), Developability Index (DI), AGGRESCAN3D, and AggScore are examples of structure-based prediction methods that depend upon 3D protein structures [119].

The SAP is one of the powerful bioinformatics approaches that predict the aggregation propensity of proteins and identify regions in a protein structure that have the potential to transform into an aggregation-prone state, using various features associated with the dynamic structural properties of proteins that assess stability and aggregation propensities [120]. The DI is a computational method that, without experimental data, enables quick screening of proteins for their tendency to aggregate. The DI may be able to predict the risk of moving forward with a specific candidate and direct protein changes to prevent aggregation [120].

AGGRESCAN3D is a sophisticated computational method designed to predict the aggregation propensities of proteins in their folded states. This method utilizes protein 3D structures as its primary input, which can be obtained from various sources, including X-ray diffraction, solution nuclear magnetic resonance (NMR) spectroscopy, or computational modeling. The structures undergo energetic minimization before analysis, ensuring that the input is suitable for accurate predictions [81]. The AGGRESCAN3D method predicts protein aggregation based on a proprietary intrinsic aggregation propensity scale for natural amino acids. It considers the aggregation propensities of each amino acid within the context of the protein’s 3D structure by modulating its intrinsic properties based on the surrounding structural context. This is achieved by focusing on spherical regions centered on every Cα carbon atom, yielding a unique aggregation score- the A3D score for each amino acid. It thus provides an atom-detailed assessment of aggregation potential, going beyond the traditional approaches that depend on linear sequence or composition-based algorithms [81].

Aggrescan4D (A4D) is a computational platform designed to predict protein aggregation, with a focus on structural context, making it a valuable tool in protein structure-related studies [121]. This computational method analyzes the solvent accessibility and physicochemical characteristics of amino acid residues in the three-dimensional framework to assess local aggregation propensities in flexible protein regions and across folded domains. The method incorporates conformational dynamics and local structural rearrangements of flexible segments into predictions [122].

A4D can predict pH-dependent aggregation in physiological conditions of proteins implicated in neurodegeneration. Because aggregation-prone states frequently correlate with temporarily exposed hydrophobic areas unreachable in static structures, its dynamic mode is crucial for capturing conformational changes. This ability is vital for proteins in neurodegenerative disease states that promote structural variability and partial unfolding. The superiority of A4D over other cutting-edge predictors, such as SolubiS and CamSol, is evident in its ability to predict the effects of mutations on aggregation in therapeutic monoclonal antibodies [121]. These findings support the utility of A4D in neurological research, particularly when mutation-driven aggregation alterations are essential, such as in familial ALS or hereditary tauopathies.

AggScore is based on three-dimensional molecular structures rather than the primary protein sequences. This structure-based approach enables more accurate predictions of aggregation propensities, especially in cases where structural variations are subtle. This method considers the characteristics of the molecular surface that contribute to aggregation, such as surface-exposed hydrophobic regions and the charge properties of nearby residues [123]. A recent advancement in structural biology, AlphaFold2, utilizes deep learning to accurately predict protein structures, paving the way for a deeper understanding of aggregation [83]. These techniques primarily consider the accessibility of protein residues and atom solvents in estimating surface hydrophobicity. The ensemble statistics are computed throughout time using brief molecular dynamics (MD) simulations in addition to the static structures at both native and misfolded forms [124].

PATH (Protein Function Annotation by Topological Heterogeneous Network) is a unique computational prediction technique that combines deep learning frameworks with domain-guided structural knowledge to enhance the accuracy of protein function annotation. PATH is a deep learning-based computational method that uses domain-guided structural knowledge to more accurately predict protein activities. This network-based deep learning framework enables PATH to extract local and global protein features, facilitating more nuanced predictions of functional annotations, especially for less-characterized proteins [125]. The “long-tail problem,” which arises from an uneven distribution of data, makes it challenging for traditional computational function annotation techniques to accurately predict infrequently annotated functions [126].

A computational method called CamSol predicts intrinsic protein solubility based on amino acid sequences and, if available, protein structure data. CamSol utilizes physicochemical characteristics to determine a solubility score, indicating the likelihood that a protein or peptide will remain soluble in physiological settings [127]. This technique combines three main algorithms: (i) calculating intrinsic solubility profiles based on amino acid sequences; (ii) applying structural corrections to account for residue environments exposed to solvent or crucial for structural integrity; and (iii) an algorithm to detect and screen potential mutations or insertions to improve solubility without sacrificing function [128]. The hydrophobicity, charge, and secondary structure propensities of amino acids (particularly α-helical and β-sheet likelihoods) are tabulated and combined to determine the intrinsic solubility profile.

CamSol can capture crucial molecular factors of solubility due to the vast protein data and biophysical analyses used in its development [129]. By separating residues involved in protein core stability from solvent-exposed residues, we may prevent harmful mutations at functionally essential sites. Protein engineering and therapeutic antibody optimization greatly benefit from CamSol’s ability to accurately predict solubility and recommend rational amino acid substitutions that increase solubility without altering the native protein fold, thanks to its dual-sequence and structure-aware methodology [127].

SolupHred is the first dedicated computational tool that predicts pH-dependent protein aggregation propensity, with a primary focus on intrinsically disordered proteins (IDPs) [130]. Unlike traditional aggregation predictors that often overlook environmental factors, SolupHred uniquely incorporates the impact of pH, a crucial variable that influences protein charge states, lipophilicity, and aggregation tendencies [130]. This approach is critical because many neurodegeneration-associated proteins are IDPs or possess intrinsically disordered regions whose aggregation behavior is significantly modulated by physiological and pathological pH variations [131]. SolupHred’s predictive ability for pH-modulated aggregation phenomena, as observed in vitro and in vivo, is demonstrated by studies that successfully replicate the aggregation behaviors of disordered proteins relevant to neurodegeneration [132]. SolupHred is used to comprehensively characterize the tendency of tau and α-synuclein to assemble in acidic or slightly changed pH conditions, which is known to occur in pathological states. That allows for insights into the environmental drivers of aberrant aggregation. This capacity is essential for understanding how diseases develop and creating pH-tuned treatment plans to prevent or reverse harmful protein buildup [132].

FoldX and CABS-Flex are robust computational tools that focus on protein structural flexibility simulations and mutation-induced changes in stability and binding energy, respectively. FoldX employs an empirically calibrated force field for thorough and energetic calculations, while CABS-Flex utilizes coarse-grained Monte Carlo dynamics for effective flexibility modeling. Collectively, they facilitate a range of research objectives, including functional dynamic studies and protein design. Their further advancement and integration hold the potential to broaden the structural biology toolkit, thereby improving our comprehension and control of protein function at the molecular level [133,134].

CORDAX is a unique technique that merges high-resolution structural data of amyloid cores with machine learning to anticipate APRs within protein sequences. In contrast to traditional methods that primarily depend on sequence characteristics, such as hydrophobicity or β-sheet propensity, CORDAX utilizes comprehensive libraries of experimentally solved amyloid fibril core structures to obtain complete three-dimensional structural information. To compute free energy estimates of interaction (ΔG), the technique first breaks down input protein sequences into hexapeptides, which are subsequently threaded onto an extensive database of 140 amyloid core structure templates using the FoldX empirical force field [133]. CORDAX is an effective tool for modeling aggregation pathways linked to various disorders because of its excellent structural fidelity and sensitivity in predicting APRs. Researchers can gain mechanistic insights into protein misfolding, polymorphic fibril formation, and aggregation kinetics that contribute to neurodegeneration by using CORDAX to identify structurally varied and previously unidentified aggregation-prone regions. Its deployment allows the identification of well-characterized APRs in pathological proteins such as tau, amyloid-β, and α-synuclein and the characterization of unconventional aggregation motifs that may represent intermediate or transient species in disease progression [135].

AmyloComp is the first program to estimate the likelihood of protein pairings co-aggregating within amyloid fibrils by examining their structural compatibilities. It focuses specifically on amyloidogenic β-arch topologies and their capacity for axial stacking [136]. AmyloComp is a computational advancement that tackles the complex phenomenon of protein-protein co-aggregation, which is increasingly recognized as a critical factor in amyloid diversity and pathological heterogeneity. That contrasts with traditional prediction algorithms that evaluate individual protein aggregation propensities [136].

Among the cutting-edge computational techniques, 2APGCNN (Two-Attribute Protein Graph Convolutional Neural Network) has shown promise in using protein structure information to predict the likelihood of protein aggregation and its consequences for neurodegenerative diseases [137]. Protein aggregation propensity is predicted from structure-based protein graphs using 2APGCNN, which leverages the capability of graph convolutional neural networks (GCNNs). In contrast to conventional sequence-based predictors, 2APGCNN incorporates a range of protein structural characteristics, including secondary structure, atomic interactions, and physicochemical properties, enabling it to capture complex spatial correlations that affect aggregation. The application of 2APGCNN extends to exploring protein aggregation across several neurodegenerative diseases. Researchers have utilized this tool to predict aggregation propensities for proteins implicated in AD (tau, amyloid-β), PD (α-synuclein), and Type 2 diabetes (islet amyloid polypeptide, IAPP) [72,138]. The accurate prediction of aggregation-prone regions facilitates the design of molecular compounds to inhibit aggregation or disaggregate existing protein clumps, thus addressing the underlying causes rather than just the symptoms [138,139] (Table 3).

## 5. Systematic Coarse-Graining Approaches for Protein Aggregation

The most basic coarse-grained approaches include lattice-based models introduced by Li et al. [157] and further developed by Vacha and Frenkel [158]. Despite their simplicity, these frameworks effectively capture essential determinants of protein aggregation [159], including the influence of nonspecific interactions on amyloid nucleation [160]. More advanced coarse-graining strategies encompass the relative entropy approach [161], multiscale coarse-graining [162], and the iterative Boltzmann inversion [163]. These systematic techniques have been widely utilized in modeling protein aggregation phenomena [164,165,166]. However, a major limitation is their dependence on accurate all-atom simulation data for parameterization [167]. One notable example of a physics-based coarse-grained model is AWSEM (associated memory, water-mediated, structure, and energy model) [168].

AWSEM models have been applied effectively to investigate protein aggregation phenomena. The AWSEM enables investigators to model the dynamics of protein-protein interactions, folding, and aggregation under specified conditions as a function of time, allowing them to make predictions concerning pathways to aggregation and the determinants that influence these processes [169]. The Bereau and Deresno model was specifically designed for the study of protein folding and aggregation, utilizing a moderate resolution of four beads per amino acid, along with implicit solvent dynamics. Thus, it enables the proper sampling of local conformations and protein behavior at physiological conditions [170]. The Bereau and Deresno model has been successfully applied in simulating protein aggregation scenarios, particularly in studying the formation of amyloid fibrils associated with various neurodegenerative diseases. Realistic aggregation pathways reproduced by the model provide evidence of its potential as a powerful tool for understanding the molecular mechanisms underlying protein misfolding and aggregation. This model has also demonstrated that the cooperative interactions among hydrophobic peptide fragments can give rise to extensive β-sheet structures, thereby accounting for a detailed representation of aggregation dynamics [170]. The OPEP (Optimized Potential for Efficient Simulation of Proteins) model employs a coarse-grained representation, simplifying the protein structure into fewer interaction sites while maintaining important characteristics of protein behavior [171]. It is particularly valuable for investigating aggregation processes and is crucial for understanding diseases related to protein misfolding, such as AD [172].

### 5.1. Molecular Dynamics Simulations in Protein Aggregation

Molecular dynamics (MD) simulations have become an essential tool for studying protein aggregation [173]. These simulations enable researchers to observe how proteins move and interact over time, either at full atomic detail or in simplified, coarse-grained formats. This ability to track molecular behavior helps uncover how proteins change shape, form early clusters, and eventually build up into larger structures, such as amyloid fibrils, processes that are often too fast, too small, or too complex to capture with experimental techniques alone [174,175].

All-atom MD simulations offer a close-up view of proteins and their surroundings, including water molecules, ions, and other biological components [176]. This MD simulation helps examine the early stages of aggregation, such as the formation of β-sheets or the role of specific amino acids in stabilizing or disrupting protein assemblies. However, these simulations are computationally demanding and typically limited to small systems and short timeframes, which can make it hard to follow the full course of aggregation [177,178].

To overcome these challenges, coarse-grained (CG) models simplify the system by grouping atoms into larger, more manageable units. This approach enables researchers to simulate larger systems over extended periods, allowing them to study the behavior of systems on a larger scale, such as fibril growth, phase separation, and the dynamics of intrinsically disordered proteins [179]. One of the most widely used CG frameworks is the Martini force field, with its latest version, Martini 3, offering improved accuracy and flexibility, particularly for systems involving membranes or complex protein interactions [180]. Beyond CG and atomistic models, hybrid approaches like AWSEM and OpenAWSEM combine the strengths of both, enabling simulations that capture broad structural changes while still resolving important molecular details. The integration of machine learning into MD workflows has also opened new doors, helping to refine force fields and improve sampling efficiency [168]. MD simulations have proven invaluable for exploring how mutations, chemical modifications, and environmental factors, such as pH or temperature, influence aggregation. They provide a dynamic and predictive platform for testing ideas, supporting experimental findings, and guiding the development of molecules that can prevent or reverse harmful aggregation. The integration of machine learning into MD workflows e.g., ML-derived CG potentials and enhanced sampling methods has improved force-field refinement and sampling efficiency, enabling more accurate and faster exploration of mutation effects, post-translational modifications, and environmental influences [181].

Coarse-grained (CG) modeling has become a widely used approach for studying protein aggregation, particularly when simulating large-scale molecular events that are beyond the reach of all-atom models [72,182]. By simplifying molecular detail while retaining essential physical interactions, CG methods enable efficient exploration of processes such as fibril formation, oligomerization, and phase separation [179]. However, this reduction in complexity comes with limitations; fine structural features, such as side-chain dynamics, solvent effects, and specific residue interactions, may be lost, which can impact the accuracy of particular predictions [183]. To address these challenges, recent advancements have introduced hybrid multiscale frameworks, adaptive resolution techniques [184], and machine learning-enhanced CG models that improve both precision and flexibility.

Modern tools such as MARTINI 3 [185], AWSEM, and OpenAWSEM exemplify these innovations, offering refined force fields and enhanced sampling capabilities [186,187]. These developments have expanded the applicability of CG modeling to a broader range of systems, including intrinsically disordered proteins and membrane-associated aggregates. Acknowledging both the strengths and limitations of CG approaches is essential for selecting the most appropriate strategy for addressing specific research questions in protein aggregation [188]. The MD simulation tools has been mentioned in Table 4.

### 5.2. Thermodynamic Approaches for Protein Aggregation

The efficient computation of free energy profiles requires both coarse-grained and atomistic simulations, along with enhanced sampling methods. The predetermined collective variables’ biased sampling techniques include meta-dynamics [189] and umbrella sampling [190]. Meta-dynamics and umbrella sampling are computer simulation methods to estimate a system’s free energy and other state functions. With parallel tempering techniques, several randomly initialized system replicas are created and operated at various temperatures. This approach enhances sampling efficiency by exchanging replicas trapped in local energy minima with replicas operating at a higher temperature, thereby eliminating the need to specify collective variables [191].

### 5.3. Protein Kinetic Profiles for Aggregation

Rare events on molecular timescales regulate kinetic evaluations of many molecular processes. Multiple simulation methods have been proposed to promote sampling of the barrier between states. A few sample methods tackle kinetics by creating a set of dynamically short, parallel trajectories that improve sampling. That includes the Markov state model (MSM) formalism, which has been utilized in investigating biomolecular processes [192,193]. This strategy boosts sampling by launching parallel trajectories. The MSM provides a complementary approach to free energy evaluations, such as replica exchange or meta-dynamics, which disregard kinetics and instead sample the energy landscape.

## 6. Discussion and Prospects

Our understanding of the protein aggregation mechanisms associated with neurodegenerative diseases has significantly improved due to ongoing advancements in computational techniques. However, there are still several exciting directions that need further investigation. Enhancing multiscale modeling techniques that combine coarse-grained and all-atom simulations is a key opportunity. These combined approaches have the potential to provide a thorough understanding of the aggregation landscape across physiologically significant timescales and length scales by bridging the gap between the mesoscale structures of mature aggregates and the atomic-level details of early aggregation events. Refined force fields, creative algorithms for smooth transitions between various simulation resolutions, and improved coupling mechanisms between scales will all be necessary to increase the precision and effectiveness of these simulations [194].

Additionally, prediction models that consider the structural context of whole proteins will be developed beyond sequence-based determinants, thanks to the growing capacity of computers and machine learning approaches. This development should enable more accurate identification of aggregation-prone areas within native protein folds and significantly lower the substantial false-positive rates now associated with aggregation propensity predictions. To design therapeutic interventions more effectively, targeting the dissolution of harmful oligomeric species or maintaining benign conformations, it is imperative to emphasize structural integration that captures the complex molecular contexts driving aggregation [194].

The use of computer technologies in rational drug and inhibitor design is another noteworthy future direction. By simulating the structural and dynamic interactions between candidate small molecules or peptides and aggregation-prone proteins, scientists can rapidly screen and refine treatments that target early toxic oligomers associated with diseases such as amyotrophic lateral sclerosis, Parkinson’s, Alzheimer’s, and Huntington’s. Using molecular dynamics simulations, computational docking, and binding affinity calculations can help identify potent aggregation modulators more quickly and facilitate the translation of these discoveries into clinical treatments. Moreover, coupling computational studies with emerging experimental techniques, including advanced spectroscopy and cryo-electron microscopy, is projected to enable the validation and refinement of simulation models. This multidisciplinary collaboration will refine our mechanistic understanding of aggregate structures and aggregation pathways, consequently allowing the development of novel biomarkers and therapeutic targets. These efforts are expected to unravel the intricate mechanisms governing protein aggregation and pave the way for innovative treatment strategies to mitigate the burden of neurodegenerative diseases. Continued investments in computational resource advancement, algorithmic innovation, and cross-disciplinary collaboration will realize these goals.

## 7. Conclusions

Protein aggregation plays a critical role in both disease progression and the development of protein-based therapeutics. Its complexity presents a significant challenge, making it essential to develop reliable and efficient strategies to understand better and manage issues related to aggregation. Computational approaches are being increasingly integrated into experimental workflows, providing valuable support in predicting and analyzing aggregation behavior. As these tools continue to evolve, we anticipate the emergence of more advanced and user-friendly algorithms that combine both protein sequence and structure, becoming standard in research laboratories. These innovations are expected to streamline the design and optimization of therapeutic proteins, ultimately enhancing biomedical research and clinical applications.

## Figures and Tables

**Table 1:** Categorized protein aggregation resources, including disease-specific databases for AD, PD, HD, and ALS (accessed on 9 November 2025 for URLs).

Resource Name	Disease Focus	Description	URL
AlzData	AD	Integrates high-throughput omics data for Alzheimer’s Disease, including transcriptomics and exome sequencing.	http://www.alzdata.org
AlzBiomarker	AD	Interactive database of fluid biomarkers for Alzheimer’s Disease, including curated measurements and meta-analyses.	https://www.alzforum.org
NIAGADS	AD	Genomic data sharing platform for Alzheimer’s and related dementias, supporting large-scale genetic studies.	https://www.niagads.org
AMP-PD	PD	Longitudinal clinical and omics data relevant to α-synuclein aggregation in Parkinson’s Disease.	https://www.amp-pd.org
PDGene Database	PD	Catalogs genetic associations and variants linked to PD, including those affecting aggregation pathways.	https://www.parkinson.org/PDGENEration
HDinHD	HD	Transcriptomic and proteomic data from Huntington’s Disease models, useful for studying HTT protein aggregation.	https://www.hdinhd.org
CHDI Foundation Resources	HD	Offers datasets and tools focused on HTT aggregation and therapeutic screening.	https://www.chdifoundation.org
Target ALS Data Portal	ALS	Multi-omic datasets including transcriptomics, proteomics, and imaging data from ALS patient samples and models.	https://www.targetals.org
ALSoD (ALS Online Database)	ALS	Genetic and clinical data related to ALS, including mutations in aggregation-prone proteins like TDP-43 and SOD1.	https://www.alsod.ac.uk

**Table 3:** Protein aggregation approaches [59].

Methods	Features	Performance Metrics	System Suitability	Ref
Amyloidogenic pattern	Pattern derived from positional scanning mutagenesis experiments on amyloidogenic peptide STVIIE	Qualitative pattern-based detection	Short amyloidogenic motifs	[94]
AGGRESCAN	Aggregation propensity scale for amino acids derived from in vivo experiments on amyloidogenic proteins	Sensitivity ~85%, Specificity ~80%	Globular proteins, therapeutic design	[98]
Zyggregator	Amino acid scales for α-helix and β-sheet formation, hydrophobicity and charge, hydrophobic pattern, and presence of Gatekeeper residues	Balanced accuracy ~80%	Proteome-wide aggregation screening	[107]
Pafig	41 physicochemical properties of amino acid	Accuracy ~82%	Sequence-based aggregation prediction	[140]
PAGE	Aromaticity, β-sheet propensity, charge, polar-nonpolar surfaces, and solubility	Not benchmarked	Peptide-level aggregation analysis	[141,142]
WALTZ	PSSM, physicochemical properties, position-specific pseudo energy terms	Specificity ~90%, Sensitivity ~70%	Short peptide amyloid prediction	[108]
AbAmyloid	Amino acid composition, dipeptide composition, and physicochemical properties	Accuracy ~85%	General amyloidogenic region detection	[143]
FoldAmyloid	Packing density and hydrogen bond probabilities obtained from protein structures	MCC ~0.72, Accuracy ~83%	Amyloid-forming proteins and peptides	[144]
SALSA β-Strand Contiguity (β-SC)	β-strand propensity	Not benchmarked	β-sheet-rich amyloid structures	[78]
APPNN	7 amino acid physicochemical and biochemical properties	Accuracy ~87%	Sequence-based prediction	[101,103]
Amylogram	17 amino acid properties such as size of residues, hydrophobicity, solvent accessible surface area, frequency of β-sheets, contactivity, and contact site propensities	Accuracy ~84%	Peptide-level amyloid prediction	[145]
ANuPP	Atom compositions of peptides and protein segments	Not benchmarked	Structural fragment analysis	[109]
TANGO	Segmental β-sheet probability derived from empirical and statistically derived energy functions	AUC ~0.82, Precision ~78%	Intrinsically disordered proteins	[113]
SecStr	Secondary structure preferences	Not benchmarked	Structural motif analysis	[114]
NetCSSP	Residue interactions and solvation energies computed using AMBER force-field.	Included in AMYLPRED2 ensemble	Sequence-based aggregation prediction	[102]
Archcandy	Scoring function derived for steric tension, electrostatic interactions, packing, and hydrogen bond formation	Not benchmarked	Structural amyloid motif detection	[146]
BetaSerpentine	β-arches (β-strand-loop-β-strand motif from Archcandy), compatibility of β-arches, compactness	Not benchmarked	β-arch motif analysis	[147]
BETASCAN	Pairwise probability tables to identify hydrogen bond-forming residues in strand pairs	Not benchmarked	Strand-pair amyloid prediction	[148]
AmyloidMutants	Potential energy scoring function derived from observed residue/residue interactions in PDB	Included in AMYLPRED2 ensemble	Mutation impact on aggregation	[149]
STITCHER	Scoring function addressing enthalpic and entropic changes in protofibril formation and BETASCAN strand pair predictions	Not benchmarked	Protofibril formation modeling	[150]
PASTA 2	Hydrogen-bonding energy functions for residue pairs derived from β-strand structures	AUC ~0.85, F1-score ~0.81	Amyloidogenic sequence screening	[89]
GAP	Residue pair potentials derived from hexapeptide sequences	Not benchmarked	Short peptide aggregation analysis	[116]
FISH Amyloid	Residue cooccurrence matrix derived from amyloidogenic and non-amyloidogenic peptides of length (4–10)	Accuracy ~83%	Peptide-level aggregation prediction	[151]
AgMata	Statistical potentials derived for residue position, secondary structure probabilities, and interaction energies	Accuracy ~86%	Sequence and structure-based prediction	[152]
3D PROFILE (ZipperDB)	Microcrystal structure of the NNQQNY peptide and atomic-level potential ROSETTADESIGN	Qualitative scoring	β-sheet segment prediction	[64]
Pre-Amyl	Template ensemble obtained from microcrystal structures of the NNQQNY peptide and KBP, atomic distance-dependent knowledge-based pairwise residue potentials	Not benchmarked	Template-based amyloid prediction	[153]
CORDAX	Thermodynamic stability calculated by threading over 140 amyloid fibril cores	Not benchmarked	Fibril core stability modeling	[154]
PATH	Modeller Dope score and Rosetta (REF15) energy values from homology models of 7 template structures	Not benchmarked	Homology-based aggregation modeling	[155]
AMYLPRED2	Consensus predictor includes outputs from AGGRESCAN, NetCSSP, AmyloidMutants, Pafig, Amyloidogenic Pattern, SecStr, Average Packing Density, TANGO, Beta-strand contiguity, WALTZ, Hexapeptide Conformational Energy.	Accuracy ~88%, Sensitivity ~85%	Broad-spectrum amyloid prediction	[103]
MetAmyl	Consensus predictor that includes PAFIG, SALSA, WALTZ, and FoldAmyloid	Accuracy ~86%	Ensemble-based prediction	[104]
SAP	Residue hydrophobicity, solvent accessible area over time obtained from MD	Not benchmarked	MD-based aggregation risk assessment	[104]
Developability Index	SAP and PROPKA values	Not benchmarked	Biotherapeutic developability screening	[156]
AggScore	Hydrophobic and hydrophilic patches obtained by using atom partial charges and logP values	Not benchmarked	Surface aggregation risk in biologics	[123]
AGGRESCAN3D 2.0	AGGRESCAN residue score, exposed surface area, FoldX energy-minimized protein structure, or Ensemble from CABS-flex simulations	AUC ~0.85, Precision ~82%	Folded proteins, therapeutic protein design	[81]

**Table 4:** Comparison of MD simulation tools and force fields for protein aggregation, highlighting resolution, features, and application scope.

Simulation Tool	Resolution	Core Features	Aggregation Suitability
GROMACS	All-atom	High-performance MD engine; supports multiple force fields (e.g., AMBER, CHARMM)	Early aggregation events, folding pathways, and solvent interactions
NAMD	All-atom	Scalable parallel simulations; long timescale modeling	Amyloid fibril growth, protein-protein interactions
Desmond	All-atom	Optimized for speed; integrated with Schrödinger suite	Drug-protein aggregation, therapeutic screening
LAMMPS	Atomistic/CG	Highly customizable; supports hybrid simulations	Aggregation in complex or heterogeneous environments
Martini 3	Coarse-grained	Refined mapping; improved protein-lipid and protein-protein interactions	Large-scale aggregation, phase separation, and membrane systems
AWSEM	Coarse-grained	Physics-based energy terms; folding and aggregation modeling	Intrinsically disordered proteins, conformational transitions
OpenAWSEM	Hybrid CG/Atomistic	GPU-accelerated; multiscale modeling capability	Aggregation with structural transitions
CABS-flex	Coarse-grained	Ensemble generation; flexibility modeling	Aggregation-prone regions, conformational sampling
CHARMM	All-atom	Versatile force field; detailed protein and solvent modeling	Mutation effects, aggregation kinetics
AMBER	All-atom	Accurate protein dynamics; widely used in folding and binding studies	Early-stage aggregation, residue-level interactions

## Data Availability

No new data were created or analyzed in this study. Data sharing is not applicable to this article.
